# Six Functions of Respiration: Isn’t It Time to Take Control over ROS Production in Mitochondria, and Aging Along with It?

**DOI:** 10.3390/ijms241612540

**Published:** 2023-08-08

**Authors:** Vladimir P. Skulachev, Mikhail Yu. Vyssokikh, Boris V. Chernyak, Armen Y. Mulkidjanian, Maxim V. Skulachev, Gregory A. Shilovsky, Konstantin G. Lyamzaev, Vitaliy B. Borisov, Fedor F. Severin, Victor A. Sadovnichii

**Affiliations:** 1 Belozersky Institute of Physico-Chemical Biology, Lomonosov Moscow State University, 119991 Moscow, Russia; skulach@belozersky.msu.ru (V.P.S.); mikhail.vyssokikh@gmail.com (M.Y.V.); bchernyak1@gmail.com (B.V.C.); maxims@mitotechpharma.com (M.V.S.); gregory_sh@list.ru (G.A.S.); lyamzaev@gmail.com (K.G.L.); severin@belozersky.msu.ru (F.F.S.); 2 Department of Physics, Osnabrueck University, D-49069 Osnabrueck, Germany; armen.mulkidjanian@uni-osnabrueck.de; 3 Institute of Mitoengineering, Lomonosov Moscow State University, 119991 Moscow, Russia; 4 Faculty of Biology, Lomonosov Moscow State University, 119234 Moscow, Russia; 5 Kharkevich Institute for Information Transmission Problems of the Russian Academy of Sciences, 127051 Moscow, Russia; 6 The “Russian Clinical Research Center for Gerontology” of the Ministry of Healthcare of the Russian Federation, Pirogov Russian National Research Medical University, 129226 Moscow, Russia; 7 Faculty of Mechanics and Mathematics, Lomonosov Moscow State University, 119991 Moscow, Russia; rector@rector.msu.ru

**Keywords:** respiration, mitochondria, reactive oxygen species, cardiolipin oxidation, aging

## Abstract

Cellular respiration is associated with at least six distinct but intertwined biological functions. (1) biosynthesis of ATP from ADP and inorganic phosphate, (2) consumption of respiratory substrates, (3) support of membrane transport, (4) conversion of respiratory energy to heat, (5) removal of oxygen to prevent oxidative damage, and (6) generation of reactive oxygen species (ROS) as signaling molecules. Here we focus on function #6, which helps the organism control its mitochondria. The ROS bursts typically occur when the mitochondrial membrane potential (MMP) becomes too high, e.g., due to mitochondrial malfunction, leading to cardiolipin (CL) oxidation. Depending on the intensity of CL damage, specific programs for the elimination of damaged mitochondria (mitophagy), whole cells (apoptosis), or organisms (phenoptosis) can be activated. In particular, we consider those mechanisms that suppress ROS generation by enabling ATP synthesis at low MMP levels. We discuss evidence that the mild depolarization mechanism of direct ATP/ADP exchange across mammalian inner and outer mitochondrial membranes weakens with age. We review recent data showing that by protecting CL from oxidation, mitochondria-targeted antioxidants decrease lethality in response to many potentially deadly shock insults. Thus, targeting ROS- and CL-dependent pathways may prevent acute mortality and, hopefully, slow aging.

## 1. Introduction

**Figure 1:**
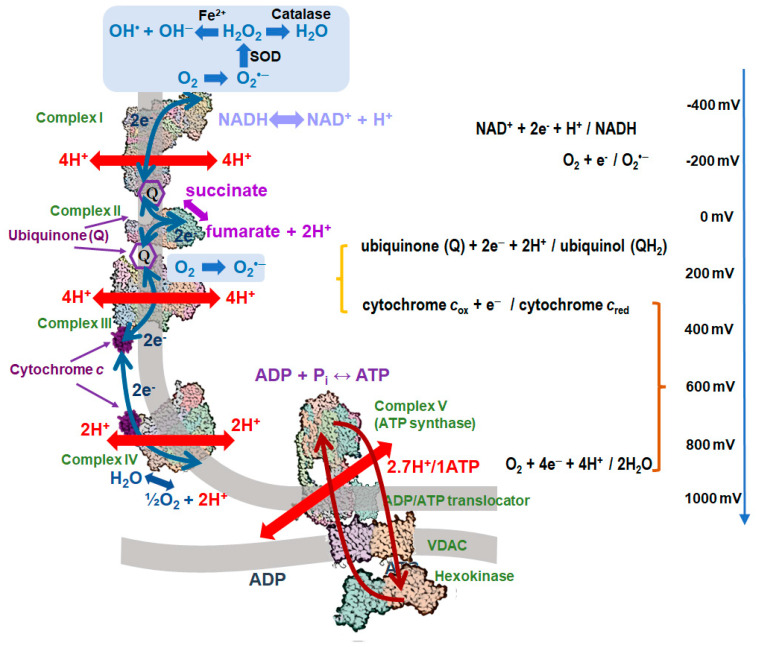
Mitochondrial respiratory chain and the complex between ATP synthase, ADP/ATP carrier, VDAC, and a mitochondrial kinase. Complexes I-IV are positioned along the scale of redox potentials on the right in accordance with their actual redox properties. The following crystal structures were used (from top to bottom): bovine Complex I (PDB ID 7R43 [4], human respiratory Complex II (PDB ID 8GS8 [5]), bovine respiratory Complex III (PDB ID 1BGY, [6]), bovine respiratory Complex IV (PDB ID 6J8M [7]), bovine respiratory cytochrome c (PDB ID 2B4Z [8]), bovine mitochondrial ATP synthase (PDB ID 6ZQM [9]), bovine mitochondrial ADP/ATP carrier (PDB ID 1OKC [10]), putative glycerol kinase-like proteins anchored on an array of voltage-dependent anion channels in the outer mitochondrial membrane of pig sperm mitochondria (PDB ID 7NIE [11]) as a model of a VDAC dimer interacting with a mitochondrial kinase. Green arrows, electron transfer steps; red arrows, proton transfer steps; orange arrows, ADP/ATP exchange. Abbreviations: SOD, superoxide-dismutase.

**Figure 2:**
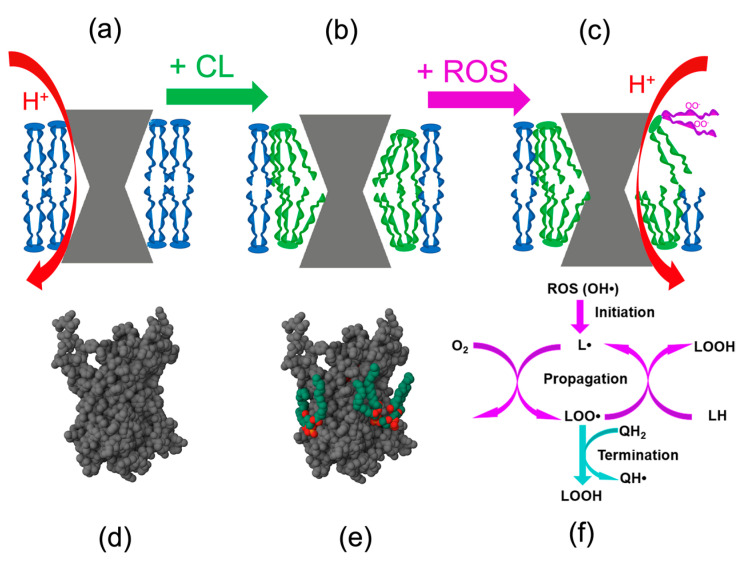
Influence of CL and its oxidation on the packing of integral membrane proteins. Top row: The scheme shows how an integral membrane protein interacts with the lipid bilayer in the presence of (**a**) cylindrical two-tail lipids, (**b**) cone-shaped four-tail CL molecules, and (**c**) after the oxidation of a CL molecule. Red arrows indicate proton leakage in cases (**a**,**c**). Bottom row: 3D structure of the bovine ADP/ATP carrier (PDB ID: 2C3E [75]) without (**d**) and with (**e**) bound CL molecules. (**f**), a scheme of a chain reaction of lipid peroxidation in the presence of an antioxidant. Abbreviations: L, lipid; LOO•, lipid peroxide; LOOH, a quenched lipid peroxide; QH_2_, a quinol-type antioxidant; see the main text and [62,72] for details.

The essence of respiration is in the oxidation of diverse substrates and the transfer of picked-up electrons to molecular oxygen (O_2_) with its reduction to water. This sequence of events is catalyzed by a series of enzyme complexes of the electron transfer chain (ETC), see Figure 1 and [1,2]. In eukaryotic cells, these are usually four ETC complexes (numbered I–IV) anchored in the inner mitochondrial membrane. Between the complexes, electrons are transferred by mobile carriers; in mitochondria, these are membrane-dissolved quinones and the cytochrome *c* protein that slides along the membrane surface (Figure 1). The ETC complexes are responsible for the stepwise transformation of redox energy into the electrochemical transmembrane proton potential (∆μ̅_H_^+^) [1,3].

The energy stored as ∆μ̅_H_^+^ is used to synthesize ATP from ADP and inorganic phosphate by ATP synthases (complexes V) [9,12]. These are rotary machines in which sequential proton transfer by so-called proteolipid subunits of the membrane-embedded oligomeric “ring” sets the rotor in motion so that three molecules of ATP are synthesized in the cytoplasmic catalytic part of the complex per complete revolution of the rotor (Figure 1) [13].

Notably, the burning of oxygen in living cells is associated with at least six different biological functions. The use of redox energy for ATP synthesis is the most important and extensive function of respiration (*function #1*). It is called oxidative phosphorylation (OXPHOS) and is responsible for the biosynthesis of approx. 50 kg of ATP per day in a healthy adult person [1]. The largest portion of redox energy, about 50 kJ/mol, is used by Complex IV, the cytochrome *c* oxidase, to pump two protons across the membrane per each electron transferred from the cytochrome *c* to O_2_; see the brown curly bracket in Figure 1 and [14].

The production of 50 kg of ATP per day implies the synthesis and subsequent consumption of comparable amounts of respiratory substrates. These reactions drive almost all cellular metabolism, which can be considered respiratory *function* #*2*.

*Function #3* of respiration, mediated by ∆μ̅_H_^+^, is to provide energy for the “electrophoretic” transport of various solutes (ions, metabolites, etc) across the membranes [1]. In prokaryotes, the potential-driven ion transport even powers the rotation of electric motors of bacterial flagella [15,16,17].

The next function of respiration is to dissipate the energy stored as ∆μ̅_H_^+^ via the futile leakage of protons through the mitochondrial membrane (*function #4*). This process is called the uncoupling of oxidative phosphorylation [18]. In thermogenic brown fat tissue, the uncoupling is executed by specific uncoupling proteins (UCP) and provides for the warming of warm-blooded animals after hibernation [19]. In some other tissues, uncoupling is mediated by transmembrane cycling of free fatty acids facilitated by various transport proteins [20,21,22].

Another function (*function #5*), called respiratory protection, removes oxygen by reducing it to water. This function was originally identified in nitrogen-fixing bacteria [23] and later extended to all breathing organisms [24]. Owing to respiration, the oxygen level is kept low inside living beings, which minimizes potential oxidative damage. Furthermore, the conversion of O_2_ into water by Complex IV (cytochrome *c* oxidase), a complex, four-electron reaction [25,26], can be considered a specific mechanism preventing the formation of poisonous reactive oxygen species (ROS).

And still, ROS, such as superoxide anion O_2_^•−^ potentially convertible into extremely dangerous hydroxyl radical OH^•^, can be generated by some of the ETC components in Complex I [27] and Complex III [28], see Figure 1. In mitochondrial Complex I, particularly large amounts of ROS are generated as byproducts of ∆μ̅_H_^+^-dependent reverse electron transfer (RET) from the ubiquinone pool to NAD^+^; this reaction generates NADH [27,29,30,31].

This brings us to respiration *function #6*, namely, the intracellular signaling of mitochondrial malfunction via increased generation of mitochondrial ROS (mtROS) in the ETC [24,32]. This *6th function* of respiration is the main topic of this review. The mtROS signaling interferes with other important intracellular signaling pathways and initiates reactions that aim to diminish the possible damage from mitochondrial malfunction [24,33,34,35,36]. However, these reactions, specifically in response to acute stress or trauma, can be excessive and even fatal, resulting in the death of the organism; see [37] for a review.

Here, we discuss how such deaths can be prevented and aging, hopefully, delayed by modulating signaling pathways related to mitochondrial ROS production in Complex I, i.e., by controlling *function #6* of respiration.

## 2. Generation of mtROS in the Respiratory Chain

### 2.1. Mechanisms of Protection against the mtROS Formation in Mitochondria

Mitochondria are considered to originate from a bacterial endosymbiont of a pro-eukaryotic cell [38]. Sequence analyses have shown that mitochondria have the closest bacterial relatives among α-proteobacteria, specifically, among the members of the order Rickettsiales that unites several families of mostly obligate intracellular bacteria [39,40,41]. However, the ancestral α-proteobacterial symbiont changed greatly in the process of transformation into the mitochondrion. In particular, mitochondrial Complex I of animals acquired more than 30 extra subunits in addition to the 14 subunits it inherited from the α-proteobacterial ancestor [42].

Concurrently, the ATP synthase (Complex V) has also undergone changes. Specifically, the number of proteolipid subunits in the membrane rotor has gradually decreased. The α-proteobacterial ATP synthase from *Paracoccus denitrificans*, for which the structure is available, contains 12 proteolipid subunits in this ring [43]. However, in the yeast ATP synthase, there are only 10 subunits [44], and in animal mitochondria, there are only 8 subunits [9,12]. These changes reflect an increased efficiency of energy conversion. Animals require the translocation of only 2.7 protons to produce one ATP molecule, compared to 4 protons translocated by the ATP synthase of *P. denitrificans*. Consequently, the threshold value of ∆μ̅_H_^+^ required for ATP synthesis is significantly higher in animal mitochondria than in *P. denitrificans*. The free energy of ATP synthesis in the cytoplasm is approximately −60 kJ/mol [1,45,46]. Thus, the threshold ∆μ̅_H_^+^ for ATP synthesis can be estimated as 150 mV for *P. denitrificans* and 225 mV for animals. The value of 225 mV is quite large, and such a ∆μ̅_H_^+^ backpressure would impede Complex III (the cytochrome *bc*_1_ complex), which exploits a relatively small redox gap (indicated by the orange curly bracket in Figure 1 and [47]). Impeding the electron flow through Complex III could produce superoxide in both Complex I and Complex III. Therefore, animals have developed sophisticated mechanisms to synthesize ATP while maintaining low ∆μ̅_H_^+^ and preventing the formation of mtROS.

One such mechanism is based on maintaining a much lower ATP to ADP ratio inside mitochondria compared to the cell cytoplasm. This is achieved through the electrophoretic nature of the ADP/ATP exchange. In the presence of membrane potential, the ADP/ATP carrier strongly favors expelling ATP molecules into the cytoplasm, ensuring a low ATP to ADP ratio within mitochondria [46]. Additionally, the ADP/ATP carrier is the most abundant protein of the intrinsic mitochondrial membrane, which prevents kinetic stalling during the export of ATP into the cytoplasm [46]. As a result, the free energy of ATP synthesis inside mitochondria decreases to about 45 kJ/mol [46], corresponding to a threshold ∆μ̅_H_^+^ for ATP synthesis of about 170 mV.

Furthermore, this mechanism appears to be further enhanced by the direct interaction between the ADP/ATP carriers and mitochondrial kinases via the voltage-dependent anion channels (VDACs), which are the main metabolite channels of the outer mitochondrial membrane (as shown in Figure 1). These interactions occur in the contact sites between the outer and inner mitochondrial membranes, as depicted in Figure 1. The complexes formed by ADP/ATP carriers and VDACs interact with creatine kinase molecules in the intramembrane space, as well as hexokinase molecules that attach to VDACs from the outer side of mitochondria [11,48], as shown in Figure 1. These and other mitochondria-associated kinases can utilize ATP produced in mitochondria, regenerate ADP, and directly return it to the mitochondrial matrix via VDAC and ADP/ATP carrier without dilution in the cytosol. This process further decreases the ATP to ADP ratio within mitochondria and enables ATP synthesis at low ∆μ̅_H_^+^ levels [49,50,51]. Notably, even a slight decrease in the ratio of ATP to ADP inside mitochondria would significantly suppress the generation of mtROS by Complex I. It has been shown that mtROS generation decreases sharply when ∆μ̅_H_^+^ falls below 80% of its maximal value, without a notable decrease in the ATP yield [52]. Activation of this “mild depolarization” mechanism has been demonstrated to reduce mtROS production in rat brain mitochondria [49,50].

Further modulation of mtROS production may proceed via the uncoupling effect, as mediated either by the common transmembrane cycling of free fatty acids assisted by the ADP/ATP carriers or tissue-specific uncoupling proteins belonging to the same family of mitochondrial carriers [53] as the ADP/ATP carrier [20,54,55,56]. This phenomenon was called “mild uncoupling” and was shown to be protective under various conditions [52,54]. The disadvantage of mild uncoupling, compared to mild depolarization, is the inevitable loss of free energy due to proton leakage. As a result, more electrons should be transferred through the ETC to yield the same amount of ATP, which would increase the sheer probability of mtROS formation.

In addition to these general mechanisms, species-dependent means of preventing mtROS formations could also be involved.

Specifically, an extreme mechanism of protection was found in human and *Xenopus* oocytes [57]. Oocytes are formed before birth and remain viable in dormancy for almost an entire lifetime (up to 50 years in humans) until fertilization. Oocytes maintain mitochondrial activity in dormancy to support the biosynthesis of the main cellular components (heme, cholesterol, etc.) [58]. To protect the cell (primarily mitochondrial DNA) from the damaging effect of mtROS, Complex I is almost completely eliminated in early oocytes, mainly through preventing its assembly [57]. Consistent with Complex I deficiency, FMN levels were shown to be reduced by about 200-fold in early oocytes, while the levels of other flavin nucleotide FAD did not change significantly. These dramatic changes in mitochondrial metabolism are typical only for early oocytes, while the content and activity of Complex I return to normal in late-stage oocytes [57]. The cause and mechanisms of age-related changes remain unclear.

It is interesting to compare the regulation of mtROS in oocytes with the regulation of male germ cells. It has been shown that the metabolism of early spermatogonia, unlike oocytes, depends on oxidative phosphorylation. Thus, damage induced by mtROS appears to be more dangerous to oocytes than to sperm cells. As mitochondrial DNA is inherited from the mother, it may indicate that the primary target of mtROS is mitochondrial DNA, not the nuclear one [59].

### 2.2. Cardiolipin and the mtROS-Signaling

The mtROS formed inside mitochondria are chemically reactive and mostly short-lived. Therefore, it is thought that the increased mtROS level message is transmitted to the outside of mitochondria via cardiolipin, which thus serves as the first receptor in the mtROS-induced signaling chains [36,60,61,62,63,64].

Cardiolipin (CL) is a four-tail lipid that is predominantly present in the inner mitochondrial membrane (IMM) of animals, constituting approximately 15% of mitochondrial lipids [65]. Initially, it was suggested that CL molecules could stabilize membrane protein complexes by interacting with multiple proteins through their four tails [66]. Subsequent investigations have revealed that CL molecules are located in and around Complexes I, III, and IV, which are organized into supercomplexes known as respirasomes [67,68,69,70,71]. These respirasomes, stabilized by CL [67], can accommodate up to 200 CL molecules [68].

Elsewhere we argued that the function of conical CL molecules in the protein-stuffed coupling membranes might be to make these membranes proton-tight by complementing the hyperbolic shape typical of most membrane proteins [72]. This suggestion is supported by experimental observations [73,74]. In particular, it was shown that the mere addition of external CL to submitochondrial vesicles from mice with compromised CL biosynthesis re-coupled the vesicles and dramatically increased the efficiency of ATP synthesis [74].

A molecule of CL carries four linoleate chains (Figure 2b,c,e) [75], each usually with two unsaturated bonds. The high unsaturation level enables (barely) the incorporation of fatty acid chains into the lipid bilayer and, at the same time, makes CL particularly vulnerable to ROS. It was repeatedly shown that CL got selectively oxidized in response to oxidative [76,77] or traumatic stress [78], whereas other lipids stayed unaffected. The oxidation of CL by mtROS generates the lipid peroxide radical (CLOO**•**), which propagates through the chain reaction mechanism; see Figure 2f and [79].

Kagan and colleagues demonstrated that peroxidized fatty acid tails of cardiolipin (CL), which tend to protrude from the bilayer (refer to Figure 2c), can interact with cytochrome *c* molecules. Cytochrome *c* is involved in the electron transfer from Complex III to Complex IV at the outer surface of the inner mitochondrial membrane (IMM), as shown in Figure 1. The fatty acid chain of CL eventually inserts into the heme-binding cleft of cytochrome *c*, disrupting the methionine-iron bond and converting cytochrome *c* into a peroxidase. This enzymatic transformation leads to the oxidation of additional CL molecules (for more details, refer to reviews [60,63]). As discussed elsewhere [62,72], these reactions occur within CL patches associated with respirasomes, which may explain the selective oxidation of CL.

Peroxidized CL molecules sticking out of the bilayer affects the proton-tightness of lipid/protein interfaces so that IMM becomes leaky (Figure 2c), and the efficiency of energy conversion drops [80]. This makes it necessary to notify the entire cell of the mitochondrial malfunction, which is done through the appearance of CL molecules on the external surface of the outer mitochondrial membrane (OMM). The (oxidized) CL molecules diffuse from IMM into OMM via the contact sites, see Figure 1 and [81].

The cell response to this notification depends on the strength of the CL signal, namely on the amount of cardiolipin in the OMM and on whether this CL is oxidized [82,83]. If the signal is weak (low CL levels in the OMM), damaged mitochondria undergo mitophagy. This process is related to macroautophagy, where unnecessary cellular components are enclosed in autophagosomes. Autophagosomes fuse with endosomes and lysosomes, breaking down the cargo for recycling [84]. Macroautophagy involves different pathways to detect dysfunctional mitochondria and sequester them. In one pathway, externalized CL is recognized by microtubule-associated protein 1 light chain 3 (LC3), a key component of the phagophore [85].

When the mtROS-induced CL signal is strong (high levels of oxidized CL in the OMM), the entire cell undergoes apoptosis (programmed cell death) to minimize damage to other cells [60,86]. Apoptosis involves an orderly disassembly triggered by the appearance of specific proteins from the mitochondrial intramembrane space, such as cytochrome *c* in mammals, in the cytoplasm. Apoptosis leads to a decrease in cell number and, ultimately, cell death.

It should be emphasized that a certain level of ROS is always present in the cell and its mitochondria. In mitochondria, these ROS are produced, in addition to the respiratory chain, by some water-soluble matrix dehydrogenases [33]. There are also enzymes in the cytoplasm that can generate ROS; see [87] for a review. Consequently, both the cytoplasm and mitochondria have systems for antioxidant defense, as well as for the remodeling of peroxidized lipids, as reviewed in [83]. All of these systems exist in a certain equilibrium with each other. However, if the level of ROS rises rapidly in the event of a malfunction, the internal defense systems do not have time to adapt, resulting in acute oxidative damage to CL, which can be perceived by the cell as a warning signal.

On the level of organs and tissues, mtROS are involved in different phenomena, of which the most studied is ischemia–reperfusion injury. This condition occurs when the blood flow to an organ is interrupted and then restored. Ischemia–reperfusion injury underlies many diseases, particularly heart attack and stroke [88]. The injury is due to the sharp increase in the ROS level upon reperfusion [89]. These ROS could be produced both in Complex I and Complex III [28,31].

The mtROS are also involved in other signaling pathways [36,90,91], one of which determines the inflammatory activation of macrophages [92,93]. It has been found that pathogen-activated macrophages stimulate their mtROS production by RET through increasing the succinate oxidation and suppressing the ATP synthesis, which causes an increase in ∆μ̅_H_^+^. The mtROS signal is recognized and leads to stimulation of the production of pro-inflammatory cytokines, which are critical for the innate immune response. The mtROS are also involved in the inflammatory response of the endothelium [94,95,96], which ensures the penetration of neutrophils into the foci of tissue inflammation. In neutrophils, the mtROS production stimulates not only a moderate activation of NADPH oxidase and degranulation [97] but also a suicidal program of extracellular trap formation (NETosis) [98]. In addition, the collective behavior of neutrophils (“swarming”) mediated by the production of leukotriene B4 also depends on mtROS [99]. It should be noted that the last two functions of neutrophils can be extremely dangerous for the organism, contributing to immunothrombosis.

Another important physiological phenomenon is the sensing of the level of oxygen in the blood by carotid bodies (CB) [100]. Mitochondria in CB receptor cells are unique in that they have a much lower affinity for O_2_ (K_d_ around 40–60 mmHg) compared to other cell types (K_d_ below 5 mmHg) [101]. As a result, even under mild hypoxia, oxygen reduction in these cells drops, stimulating RET-dependent mtROS production [102]. It should be noted that mtROS generation is one of several mechanisms (both mitochondria-dependent and mitochondria-independent) of O_2_ sensing by CB.

Not surprisingly, nature uses different means to specifically constrain the mtROS formation. For instance, it was shown that the ischemia in postnatal day 10 rats causes deactivation of Complex I, thus decreasing the intensity of an eventual mtROS burst in response to reperfusion [103].

## 3. Modulation of mtROS Signaling by Mitochondria-Targeted Antioxidants

### 3.1. Prevention of Cardiolipin Oxidation

Since the strength of a mtROS-induced signal depends essentially on the extent of oxidative damage to CL, the oxidized CL should be promptly remodeled by replacing the peroxidized tail(s) [104,105].

**Figure 3:**
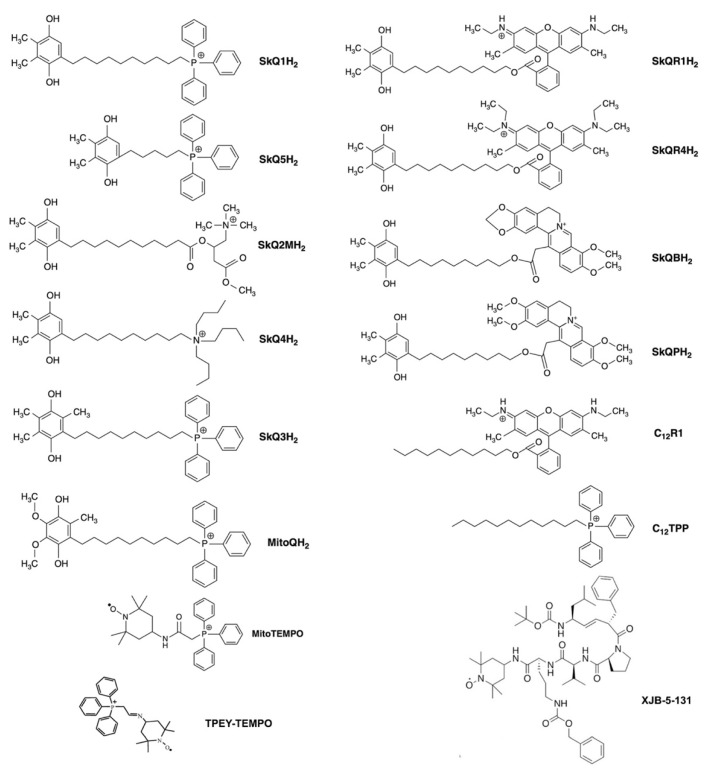
Structure of different mitochondria-targeted compounds. Adapted from [94].

In addition, the CL- signal can be modulated by protecting CL from oxidation. This could be done by using antioxidants, which can prevent the propagation of mtROS and/or quench the radical states of CL, as shown in Figure 2f. Particularly promising are mitochondria-targeted antioxidants (MTAs), which specifically accumulate in mitochondria that are negatively charged relative to the cytoplasm. Such MTAs usually carry a hydrophobically shielded positively charged moiety, which can carry the whole molecule across the mitochondrial membrane being driven by electric potential difference, see Figure 3 for structures of different MTAs and [77,106,107] for some of their applications.

And indeed, oxidation of CL upon oxidative stress could be prevented, with distinct benefits for the organisms, by adding diverse MTAs. For instance, the oxidation of CL in response to oxidative stress in mitochondria was prevented by SkQ1, where a naturally recoverable antioxidant plastoquinol moiety was linked to a penetrating cation triphenyl-phosphonium, see Figure 3 and [77].

Oxidation of CL in mitochondria was also prevented by a spin label Tempo linked to triphenyl-phosphonium ([2-(1-oxyl-2,2,6,6-tetramethyl-piperidin-4-ylimino)-ethyl]-triphenyl-phosphonium; TPEY-Tempo) [107]. As well, a trauma-induced CL oxidation in the brain could be fully prevented by XJB-5-131, a conjugate of 4-amino TEMPO and the chemically modified segment of a bacterial membrane targeting antibiotic Gramicidin S, which effectively delivered the nitroxide into mitochondria [78], see also the next section on more data on MTAs.

Importantly, all MTAs that effectively protected mitochondrial CL from oxidation were small, amphiphilic molecules. As discussed elsewhere [62,72], their amphiphilic nature likely enables them to penetrate the membrane from the aqueous phase and protect CL molecules within respirasomes. These CL molecules are typically inaccessible to natural antioxidants, such as ubiquinol and vitamin E, present in the bulk lipid bilayer.

### 3.2. Prevention of Acute Organismic Damage

In eukaryotes, the excessive production of mtROS leads, in some cases, to an over-reaction of the whole organism to the stress or damage; see [37] for a review. In many cases, such reactions end up with the death of an organism. These aberrant reactions can be rationalized by the concept of phenoptosis (programmed death of an organism) proposed by one of the authors (VPS) [22,108,109,110]. By analogy with apoptosis, the concept implies the existence of suicidal programs that eliminate a damaged or diseased individual to protect the entire population. Such programs are counterproductive for individual organisms but important for the survival and evolvability of species.

It has been hypothesized that such holistic aberrant responses can be prevented or attenuated by suppressing the formation of mtROS and/or propagation of mtROS-induced signals out of mitochondria [110]. To test this hypothesis, we designed and tested an MTA SkQ1 (Figure 3), where a naturally recoverable antioxidant plastoquinol moiety was targeted to mitochondria by penetrating cation triphenyl-phosphonium (a Skulachev-ion according to Green [111]). The subsequently obtained conjugates of different quinones with diverse penetrating cations (Figure 3) make the family of SkQ-type MTAs [77,112,113,114].

In our earlier studies back in 2008-2011, it was shown that MTAs of the SkQ family (SkQs) saved animal lives in various models [77,115]. The first success was achieved by Zorov and colleagues, who observed the prevention of death caused by kidney ischemia and reperfusion using SkQ1 and SkQR1 in a single-kidney model in rats [115]. In the same model, SkQ1 protected rats from combined ischemia-reperfusion of the kidney and intraperitoneal injection of mitochondria [116]. The same group found that treatment with SkQR1 significantly reduced mortality in neonatal rats caused by intraperitoneal administration of LPS [117] and adult rats with acute pyelonephritis caused by bacteria injected into the bladder [118]. Another group in our laboratory demonstrated the protective effect of SkQ1 in a mouse model of systemic inflammation induced by lethal intravenous injection of TNF [96]. SkQ1 and SkQR1 have been shown to suppress manifestations of brain damage, myocardial infarction, and kidney damage caused by ischemia [115], as well as symptoms of Alzheimer’s disease [119]. Therapeutic effects of SkQ1 have also been observed in mouse models of autoimmune arthritis [120] and ulcerative colitis [121]. Some MTAs prevented the oxidation of CL when added, even in nanomolar amounts [77].

Multiple positive effects of MTAs have also been described in various models by other research teams (for detailed reviews, see [37,83,122]). One of the first MTA, MitoQ, a conjugate of ubiquinone with triphenylphosphonium cation, reduced markers of acute hepatic and renal dysfunction [123] and cardiomyopathy [124] in LPS-infected rats. A stable aminooxy radical (TEMPO) conjugated to a triphenylphosphonium cation (MitoTEMPO) attenuated the sepsis-induced acute kidney injury [125], ulcerative colitis [126], LPS-induced acute systemic inflammation, and liver failure [127,128] in mouse models. It was shown that intranasal administration of MitoTEMPO suppresses pathological inflammation and reduces mortality in mice infected with influenza A virus [129].

**Figure 4:**
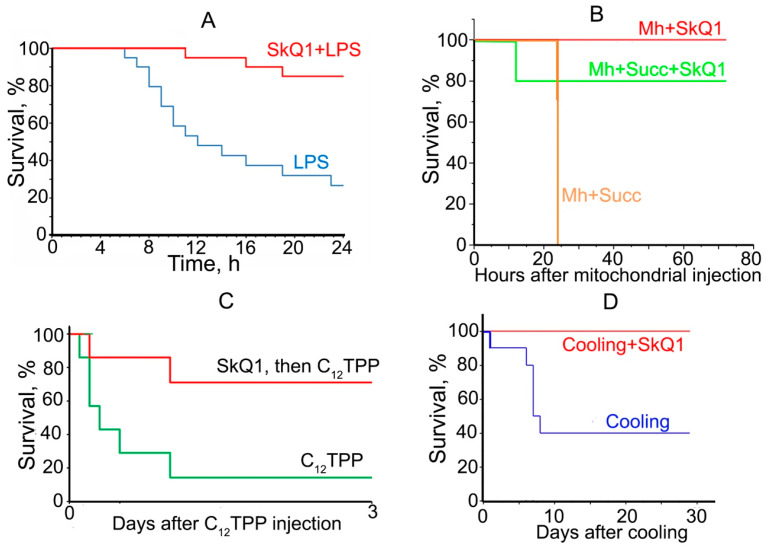
Mitochondria-targeted antioxidant SkQ1 prevents the lethal toxicity of four different shocks. (**A**) SkQ1 prevents the lethal toxicity of bacterial lipopolysaccharide (LPS). LPS at doses of 30 mg/kg was injected intravenously. SkQ1 (1.5 mmol/kg/day) was administered intraperitoneally for five days. С57Bl6 (3 months) mice were used; (**B**) SkQ1 prevents the rapid death of mice after injection of mitochondria (10 mg protein/kg body weight) into the tail vein. SkQ1 (1.5 μmol/kg) was administered intraperitoneally daily for 5 days before and after mitochondrial injection. 5 mM succinate (Succ) was added to mitochondria prior to injection. (**С**) SkQ1 protects mice against toxic doses of C_12_TPP. Survival of mice after a single intravenous injection of 34 μmol/kg C_12_TPP. SkQ1 (1.5 μmol/kg/day) was injected as in (**A**); (**D**) SkQ1 prevents mice from rapid death caused by cooling. Mice were placed at −20 °C for 1 h and then returned to room temperature. SkQ1 (1.5 μmol/kg) was administered intraperitoneally for 5 days before cooling and for 3 days after cooling. Adapted from [130].

In the most recent study [130], we analyzed the effects of SkQ1 in four completely different mouse models of fatal shock caused by (1) bacterial lipopolysaccharide (LPS), (2) injection of mitochondria into the bloodstream, (3) short-term cooling of animals at −20 °C, and (4) lethal dose of mitochondria-targeted toxin. In the first three cases, the mice were completely saved from death, and in the last one, every second mouse survived (Figure 4).

Partial prevention of LPS-induced fatal shock by an MTA treatment was reported in 2019 by Zorov and colleagues [117], who applied SkQR1 to 7-day-old newborn rats. In our recent study [130], the pretreatment with SkQ1 almost completely prevented LPS-induced mortality in young mice (Figure 4A), while in old animals (in which LPS toxicity was more pronounced), the protective effect of SkQ1 was only partial. LPS shock is thought to be associated with the so-called “cytokine storm” when large amounts of pro-inflammatory cytokines are released into the bloodstream. Pretreatment with SkQ1 stopped the increase of one of the most important cytokines, interleukin IL-6, in both young and old mice [130].

Injection of mitochondria into the bloodstream at least partially resembles sepsis because mitochondria, owing to their bacterial origin, are recognized by immune cells by the same receptors as bacterial pathogen-associated molecular patterns (PAMPs). In addition, some mitochondrial components are recognized as damage-associated molecular patterns (DAMPs), which signal the body of massive tissue damage, as occurs after severe trauma or surgery [37,131,132]. Pretreatment of mice with SkQ1 almost completely prevented mortality caused by intravenous administration of mitochondria (Figure 4B). Next, a lethal dose of the mitochondria-targeted toxin dodecyltriphenylphosphonium (C12TPP) was administered to mice, causing their death. In the group pretreated with SkQ1, 70% of mice survived (Figure 4C). In one more trial, mice were placed for one hour at −20 °C. Half of the mice died between days 5 and 8. In the group pretreated with SkQ1, all the animals survived (Figure 4D). The common trait of these four very different shock exposures was the increase in the level of pro-inflammatory cytokines IL-6 and TNF-α in the blood, which was prevented by SkQ1 [130].

More recently, a group in China independently reported the ability of SkQ1 to protect against a severe hemorrhagic shock (HS) in rats [133]. Again, SkQ1 not only prevented organ damage but also reversed the HS-induced increases in blood levels of pro-inflammatory cytokines IL-6 and TNF-α.

A critical component of many pathologies is excessive inflammation, which can be triggered by mtROS. Probably the most prominent example of this kind is the SARS-CoV2-induced coronavirus infection COVID-19, with about 7 million deaths worldwide. It is generally accepted that the pathogenesis of COVID-19 can be divided into two phases: early, when viral infection predominates, and late, when immune responses determine the severe course of the disease. In support of this finding, only a very low lung viral load was found in samples from patients who died from COVID-19 during the initial outbreak in Wuhan, China [134]. It is still unknown why in some cases the immune response gets out of control, although the infection is mostly stopped by antivirus systems. Cain and Sidlowski [135] suggested that the release of endogenous DAMPs, leading to a cytokine storm, is crucial for the severe course of COVID-19, whereas the active infection is not a direct cause of death. Their hypothesis, hence, can be reformulated as “a severe form of COVID-19 is an example of acute phenoptosis.” Further arguments in favor of this view, as well as the possible role of excessive mtROS production in the pathogenesis of severe COVID-19, are presented in our recent publications [136,137,138]. Notably, applications of diverse MTAs yielded perspective results in murine [139] and cell culture [140] models of COVID-19.

These data on the application of diverse MTAs on different types of shocks are consistent with data on the important role of mtROS and their “receptors”, such as CL, in activating the innate immune response leading to inflammation. The hyperactivation of the innate immune system may contribute to mortality and be a major target for the protective action of MTAs, see [37] for a review. Overall, these data on the possibility of preventing or suppressing diverse adverse organismic reactions by MTAs strongly support the involvement of mtROS-signaling in such cases of acute phenoptosis.

### 3.3. Aging (Chronic Phenoptosis) and Production of mtROS in Complex I

As discussed elsewhere [22,109], aging can be viewed as a chronic phenoptosis—a gradual programmed death of the organism. Therefore, the mechanisms dependent on mtROS may also play a role in aging [141,142]. An increase in mtROS production and its role in aging have been discussed for several decades [143]. However, the mtROS production in isolated mitochondria generally does not increase with age. This controversy was partially resolved by van Loon and colleagues [144], who demonstrated impaired mitochondrial sensitivity to ADP in an aging human muscle. It was shown that the hampering of ADP transport into mitochondria and the subsequent increase in ∆μ̅_H_^+^, due to the increase in the ATP to ADP ratio within mitochondria and suppression of ATP synthesis, stimulated the mtROS production, presumably through RET in complex I.

**Figure 5:**
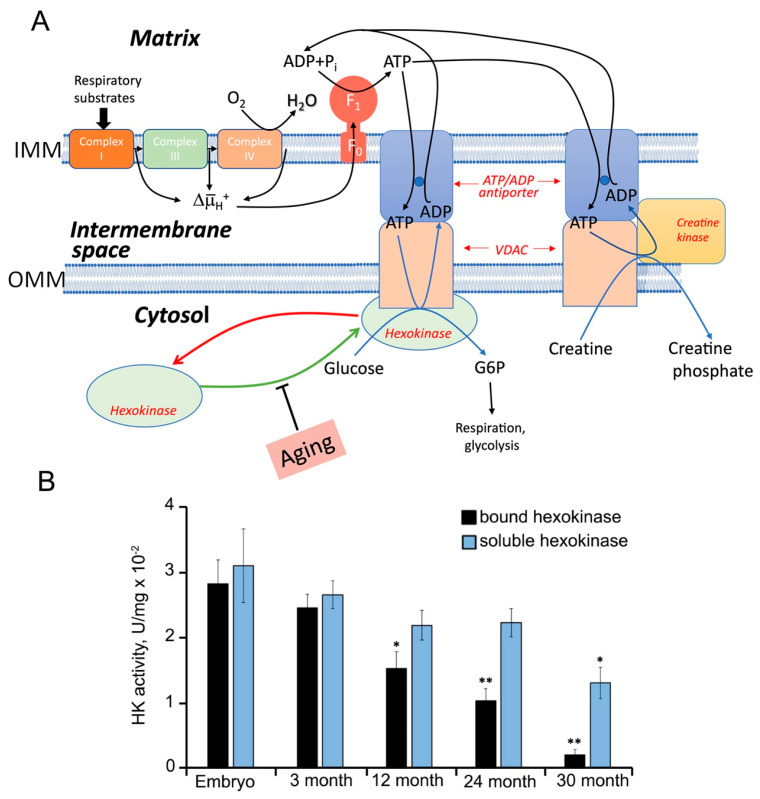
Mechanism of mild depolarization and its dependence on aging. (**A**), The interplay between the respiratory chain (Complexes I, II, III, and IV), H^+^-ATP-synthase (Complex V, also known as F_O_F_1_), the transporters of ATP and ADP (ADP/ATP carrier and VDAC), and hexokinase I or II bound to VDAC. Creatine kinase is is in the intermembrane space, contacting the outer surface of the ADP/ATP carrier. The creatine phosphate produced by this kinase is released into the intermembrane space of the mitochondrion. The hexokinase-mediated depolarization should be driven by the dissipation of some energy due to the transfer of the phosphoryl residue from ATP to glucose when G6P is formed. During aging, the level of mitochondria-associated hexokinase decreases, which prevents mild depolarization and consequently leads to increased mROS production. (**B**) Experimental data illustrating the age-dependent decline in mitochondrial-bound hexokinase activity in mouse embryos and 3-, 12-, 24-, or 30-month-old mice. * *p* < 0.01; ** *p* < 0.001 (mouse embryos, 12-, 24-, or 30-mo-old mice compared with 3-mo-old mice). Date adapted from [145].

Our recent results have uncovered the mechanism underlying the increased production of mtROS in various tissues and in various organisms upon aging [145]. We showed that aging is accompanied by a decline in the ability to decrease the mtROS production by fast channeling ATP out of mitochondria and ADP into it, thus dramatically decreasing the ATP to ADP ratio (see Section 2.1). This mechanism, which is mediated by ADP/ATP carriers and VDACs, depends on the ability of hexokinase and/or creatine kinase to bind to the surface of the outer or the inner mitochondrial membrane, respectively, and to utilize promptly and directly the synthesized ATP; see [49,50,51,145,146]. Our experiments showed that the antioxidant effect of mitochondrial kinases disappeared in skeletal muscle, diaphragm, heart, spleen, brain, and partly in the lungs and kidneys of 2.5-year-old mice, which correlated with the dissociation of kinases from mitochondria see Figure 5. The same age-related decline in the level of related kinases was observed in human muscle but not in long-lived mole rats and bats. It was concluded that the association of hexokinase and creatine kinase with mitochondria might be an essential component of protection against aging [145].

Also, our recent results shed some light on mechanisms underlying the age-dependent dissociation of hexokinase and creatine kinase from mitochondria. Earlier, it was shown that CL is tightly incorporated into the protein structure of the ADP/ATP carrier (see Figure 2e and [147]) and is essential for its conformational changes during nucleotide transport [56]. Oxidation of CL disturbs its interaction with the ADP/ATP carrier, which may affect the formation of a complex with VDAC and, consequently, the binding of hexokinase or creatine kinase to mitochondria [148,149]. Normally, the oxidized CL is promptly recovered by its remodeling to maintain a constant composition of its acyl chain with a high ratio of n3/n6 polyunsaturated fatty acids (PUFA). In young organisms, this mechanism helps overcome stress and contributes to post-stress adaptation [150]. We showed that the efficiency of CL remodeling decreased in older organisms [105]. The complex mechanism of CL remodeling includes monolysocardiolipin as the main intermediate. Its degradation to di-lysocardiolipin in the case of slower remodeling may be fraught with the formation of toxic peroxidation products [104]. The decrease in the intact CL with age, in turn, may affect the fine-tuned interaction between the ADP/ATP carriers, VDACs, and mitochondrial kinases. Specifically, the impediments in CL remodeling may prompt the dissociation of hexokinases from mitochondria. As a result, the ATP to ADP ratio within mitochondria should increase with all the adverse consequences, including higher steady state ∆μ̅_H_^+^ levels and elevated mtROS generation.

Apparently, aging deteriorates the turnover of CL in mitochondria, which can have multiple consequences for the efficiency of energy conversion, mtROS generation, and the reliability of CL-involving signal chains. A disbalance of all these systems might result in increased metabolic fluctuations and more frequent cases of transient ischemia. Not surprisingly, the risk of ischemia/reperfusion damage was shown to increase with age [151].

As mentioned, SkQ1 efficiently prevented the oxidation of mitochondrial CL [112,130,152]. Consistently with these results, SkQ1 prevented the decrease of CL content during premature aging in mtDNA mutator mice [153].

Last but not least, SkQ1 also prevented the involution of the thymus, a specialized primary lymphoid organ of the immune system where T cells mature. In humans, thymic cellularity reaches its peak during the first year of life and then undergoes a many-fold decrease, which is thought to reflect the decrease in immune capacity and to correlate with aging; see [154] for a review. Specifically, 250 nmol/kg of SkQ1 suppressed the age-related thymic involution of spleen follicles (where B lymphocytes are produced) in normal and premature aging (OXYS) rats [155].

All these findings indicate that the basic mechanisms underlying the overproduction of mtROS and leading to mortality after fatal shocks (acute phenoptosis) may overlap with mechanisms of aging (slow phenoptosis).

## 4. Conclusions and Outlook

The benign effects of structurally diverse MTAs on various types of shock exposure described here, as well as aging, point to the possibility of preventing different adverse outcomes by inhibiting ROS-induced and CL-mediated signaling circuits. These results indicate that MTAs could be a promising weapon in the fight against various diseases and aging, thus making it possible to take respiratory function #6, namely the ROS signaling, under human control.

## Data Availability

Data sharing not applicable.
